# The Role of Caregivers in Promoting Connectedness in Youth with Mental Health Concerns

**DOI:** 10.3390/children11121469

**Published:** 2024-11-30

**Authors:** Brody Andrews, Govind Krishnamoorthy, Vicki C. Dallinger, Darryl Maybery

**Affiliations:** 1 School of Psychology and Wellbeing, University of Southern Queensland (UniSQ), Toowoomba, QLD 4350, Australia; u1043383@umail.usq.edu.au; 2 Manna Institute, Center for Health Research, University of Southern Queensland (UniSQ), Toowoomba, QLD 4350, Australia; govind.krishnamoorthy@unisq.edu.au; 3 School of Rural Health, Monash University, Melbourne, VIC 3800, Australia; darryl.maybery@monash.edu

**Keywords:** caregivers, youth, youth recovery, mental health, connectedness, CHIME

## Abstract

Background/Objectives: Mental health concerns among youth represent a critical global public health issue. Research has found that youth with mental health concerns are often reliant on their caregivers while being isolated from peers. Guided by the recovery model of mental health care, this study investigates the often-overlooked role of caregivers in fostering ‘connectedness’ in youth; Methods: Semi-structured interviews were conducted with nine caregivers of youth with mental health issue; Results: Thematic analysis underscored three tasks in the role of caregivers in promoting connectedness in youth: (a) understanding the nature and quality of social networks, (b) supporting readiness to engage in social relationships, and (c) promoting their youth’s social problem solving and self-efficacy; Discussion: These tasks highlight the complex dialectics faced by caregivers in supporting youth recovery. The findings hold key implications for developing interventions, resources and policies designed for caregivers.

## 1. Introduction

The World Health Organisation [1,2] reports that mental health extends beyond the mere absence of illness or disability. It significantly influences an individual’s ability to navigate life’s challenges, pursue personal goals, and contribute positively to their community. In Australia, there has been an alarming increase in the diagnosis of mental health issues among young people [3]. Almost 40% of individuals aged 16 to 24 were diagnosed with a mental health issue, significantly higher than other age groups [3]. This trend is not isolated to Australia; its prevalence is increasing globally [4].

While traditional population-based strategies have prioritised promoting wellbeing and symptom reduction, these approaches often lack the specificity required for youth recovering from mental health concerns [5]. For example, the quality of relationships significantly affects youth coping and wellbeing [6] but youth with mental health concerns often face unique challenges in initiating and maintaining relationships and are more susceptible to social isolation [7]. Social isolation, characterised by limited and low-quality contact with others, is a pervasive issue that substantially impacts the youth population and their sense of connectedness [8].

The importance of connectedness, or lack thereof (social isolation), is especially evident when considering its effects on youth mental health and its role in recovery. Youth are particularly susceptible to the detrimental effects of social isolation due to their ongoing development and dependence on social interactions for emotional and social growth. Significantly, social isolation is strongly linked to mental health issues and often results in decreased feelings of connectedness among youth [9,10]. The COVID-19 pandemic exacerbated this, with research [11] indicating a rise in psychological distress correlating with increased social isolation. The isolation experienced can stem from various factors, such as missed chances for incidental social interaction [12] and the stigma and self-stigma associated with mental health issues [7,13]. Youth dealing with mental health problems often find themselves increasingly isolated due to school absenteeism, which limits their social interactions [12]. This sense of isolation is further compounded by the combination of social skills deficits and the disruptive effects of mental health issues on developmental capacities, creating a vicious cycle of isolation [14,15].

Many young people with mental health concerns rely upon caregivers for support. A caregiver is somebody who performs “the nurturing, tasks, resources, and services that meet the day-to-day needs of children and youth with special health care needs at home” [16]. Caregivers typically spend a significant amount of time providing unpaid support to family or friends who at times experience difficulty with self-care [16]. This role has been considered a crucial component of quality mental health service delivery [17] and are recognised worldwide in government policy and rhetoric [18,19,20,21]. Emotional and practical support is often provided by caregivers including communication and navigation assistance in the complex healthcare systems and life more broadly. Those caring for youth also significantly influence the social skills and psychosocial competence of youth with mental health concerns [11,12,22]. Despite the established influence of caregivers’ support on feelings of isolation and psychosocial abilities, the literature lacks a comprehensive understanding of the specific role caregivers play in promoting social connectedness among youth with mental health concerns [18,19,23].

Many studies underscore that a sense of connectedness to school, community, and family can protect against social isolation and various mental health issues in youth [6]. Further analyses have shed light on how robust social bonds can fortify resilience and support positive developmental outcomes [22,23]. Arango et al., 2019 [18] support this view by showing that connectedness can counterbalance the negative impacts of adverse childhood experiences on mental health, emphasising its essential role in youth recovery. Recent research suggests that supportive relationships, including perceived social support from peers, teachers, family, and friends, have a profound protective effect on mental health and can significantly enhance psychological and academic outcomes in young people [19,20].

**Figure 1:**
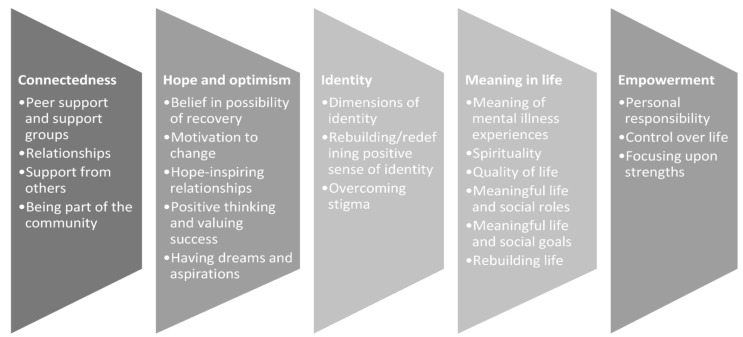
CHIME framework of personal recovery [31].

Given the considerable burden of mental health issues on young people, their families, and health services, it is crucial to explore methods to promote connectedness among youth with mental health concerns [21]. Personal recovery, or recovery, is different to clinical recovery and originates from the model of adult psychiatric rehabilitation and embodies a humanistic theme through the promotion of self-efficacy and maximising potential [24]. In their broadly disseminated and evaluated framework of personal recovery, Leamy et al. [25] devised a model for adult personal recovery identifying five interrelated processes: connectedness, hope and optimism, identity, meaning and purpose, and empowerment (CHIME) that has served as the foundation for more recent recovery-orientated interventions within adult mental health. The CHIME personal recovery model underscores the importance of personal and social resources and the value of supportive relationships (see Figure 1). This model has become the accepted paradigm in mental health services and is recognised as the best practice worldwide [25,26,27,28,29]. In Australia, mental health service provision has prioritised the personal recovery paradigm, as outlined by the National Framework of Recovery-Oriented Mental Health Services [30] and the Mental Health Coordinating Council [31].

**Figure 2:**
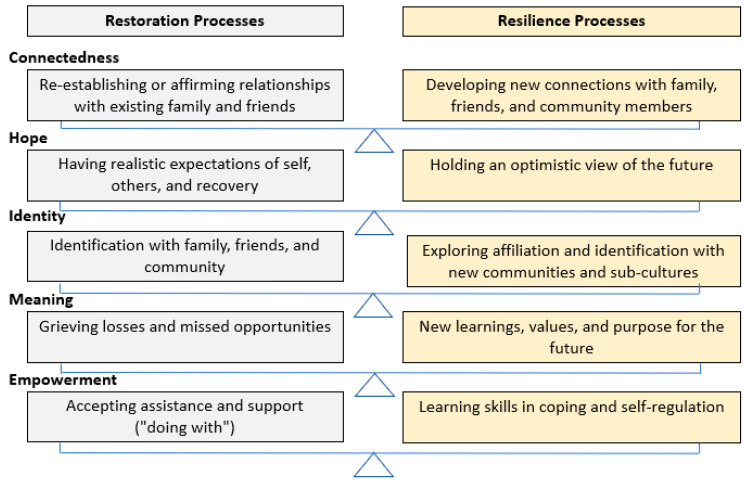
Restoration and resilience processes in youth recovery [36].

While traditional population-based strategies have prioritised promoting wellbeing, these approaches often lack the specificity required for youth recovering from mental health concerns [5]. For example, the quality of relationships significantly affects youth coping and wellbeing [6] but youth with mental health concerns often face unique challenges in initiating and maintaining relationships and are more susceptible to social isolation [7]. The CHIME framework has shown efficacy in many adult recovery applications. However, research has shown that when applied to youth recovery a more developmentally appropriate and ecological view is required [32,33,34,35]. A recent youth recovery conceptualisation (see Figure 2; [32]) has assessed the importance of existing frameworks including CHIME and through multisystemic analysis identified additional processes of restoration and resilience. These additional processes note that connectedness includes the restoration of relationships as well as the development of new connections related to mental health and healthy identity development.

The existing research underscores the importance of caregiver support, psychological control, and behavioural control in youth social development [36]. However, the application and outcomes of these dimensions of caregiving can be complex and multifaceted, varying based on factors like developmental stage, cultural context, and individual differences among youth. While the current body of literature provides valuable insights into the general role of caregivers, it does not adequately explore the specific roles and strategies that caregivers employ when supporting youth with pre-existing mental health challenges [34]. Following on from the work of Dallinger et al. [32], this research explores the need for connectedness in a more detailed analysis. Understanding these specific roles is crucial for developing targeted interventions and support systems that cater to this population’s unique needs and challenges. A thorough understanding of how caregivers can promote connectedness in young individuals dealing with mental health concerns is crucial to recovery [37,38,39].

### Aim and Research Questions

This study forms a key element of a broader research program focused on youth recovery. It aims to aid caregivers of youth with mental health concerns. Connectedness constitutes a crucial element in youth mental health recovery, and the existing literature on the role of caregivers in this context requires further exploration. The objective of the present study is to advance the exploration and comprehension of the part caregivers assume in youth mental health recovery by addressing the research question:

1. What is the role of caregivers in promoting connectedness for youths with mental health concerns?

## 2. Materials and Methods

### 2.1. Recruitment and Participants

The study received ethical approval from the university ethics committee (H20REA100). It sought to explore the lived experiences related to the roles and needs of caregivers in youth mental health recovery. Purposive sampling was employed, selecting primary caregivers for youth aged 12–24 who have previously engaged with mental health services. This research focuses on the youth population aged 12 to 24 years, as there is still much controversy surrounding the concept of recovery for younger children [40,41,42]. Demographic information of youth in care are provided in Table 1.

Recruitment included personal communication, email outreach, and flyer distribution within Queensland local mental health networks and consumer advocacy groups. Prospective participants initiated contact and were then provided with an information pack via email, including a consent form and interview question guide. Participation was voluntary, with potential incentives of retail vouchers awarded through a random draw. All participants identified as parents of youth in care and were Queensland residents; demographics are included in Table 2.

### 2.2. Data Collection

The data collection process was conducted through semi-structured interviews across 6 months, during 2021. Four interviews were conducted via telephone, the remainder were face-to-face. The duration of the interviews varied from 35 to 65 min, reflecting the level of participant engagement and the depth of responses. Each interview included the collection of demographic information, followed by a series of open-ended questions developed by the research team targeting their experience of recovery processes in relation to their dependents experience with mental health services and youth recovery (see File S1). These questions were designed to probe deeper into the participants’ experiences and viewpoints, generating rich, detailed, and nuanced data. All interviews were digitally recorded, transcribed thoroughly, and reviewed for accuracy. Data saturation determined the sample size.

### 2.3. Data Analysis

Reflexive Thematic Analysis (TA) was employed to identify meaningful patterns across the dataset, leveraging the researcher’s unique perspective as a resource rather than a potential source of bias [43]. Data analysis was conducted using NVivo version 1.7.1 [44]. A hybrid deductive and inductive methods design was used to facilitate a comprehensive analysis. The CHIME framework [25] guided the deductive interpretation, whereas the inductive analysis allowed the data to generate themes uninfluenced by pre-existing theories [45]. The recordings were listened to several times, while transcribed data and field notes were read, re-read, and checked against recordings for accuracy. The coding of data was conducted by two researchers. Each researcher worked individually to encode all information and produce themes and sub-themes. The coding of data and themes was then reviewed collectively in an iterative process until consensus was met between researchers on key themes and sub-themes.

### 2.4. Reflexivity

The research methodology incorporates reflexivity to enhance transparency in the analysis process and to manage potential biases or assumptions from the researcher. All authors acknowledge previous work with youth and the potential risk of confirmation bias is present—that data may corroborate professional experiences and neglect contradictory evidence. Moreover, additional layers of complexity arise from identity which may contrast with the experiences of the female participants. This distinction necessitates consideration, as societal or cultural norms, personal experiences, and perceptions can shape shared narratives and influence interpretations.

## 3. Results

The TA revealed three themes in the role of caregivers in promoting connectedness in youth: (a) understanding the nature and quality of social networks, (b) supporting readiness to engage in social relationships, and (c) promoting social problem-solving and self-efficacy. These themes collectively depict the diverse strategies and challenges caregivers face in fostering social connectedness among youth with mental health issues (see Table 3).

### 3.1. Theme 1: Understanding the Nature and Quality of Social Networks

Caregivers spoke of the value of being aware of the youth’s relational context within and outside the family and across different settings (e.g., online). The caregiver interviews revealed insights into their understanding of their youths’ social networks and their challenges. For instance, Caregiver 6 expressed a view indicating their youth’s desire for more friends and a peer group of their age:

“She’s only got one friend. So, she’s wanting to get more friends and wanting to make a group that she can be with and feel a part of.”

[CAREGIVER 6]

This nuanced understanding reflects caregivers’ recognition of their youth’s social developmental needs. It emphasises the critical role of interpersonal relationships in a youth’s life, aligning with the assertion that social bonds significantly impact an individual’s mental health and wellbeing [46]. Moreover, it highlights caregivers’ crucial role in supporting their youth in developing friendships. For instance, this and other studies have found that caregivers’ approval and support of friendships can positively influence the quality and stability of these relationships [47]. Some caregivers also acknowledged their child’s reluctance to form social connections and the need for encouragement and scaffolding to build and maintain relationships. Caregiver 3 mentioned:

“She didn’t want to connect… even when she was here, she was pretty resistant to connecting…”

[CAREGIVER 3]

For online interactions, caregivers spoke of the need to be aware of engaged platforms, how they use these platforms, and their perceptions of belonging, acceptance, and social inclusion in their online interactions. Caregiver 4 stated:

“Well, he has a social media account, like he’s got an Instagram account and Facebook, but he doesn’t really talk to people on it… He doesn’t connect with people on it.”

[CAREGIVER 4]

The caregiver’s statement underscores their awareness of the youth’s limited engagement in social interactions on social media platforms. Caregiver 8 shared a concern about their youth’s usage of Snapchat and peer pressure:

“Oh, yeah, he is [on Snapchat]. It doesn’t help with the anxiety and the peers and the overall drinking and smoking and his friends putting photos up of them vaping. You know, so that whole peer pressure, which he’s very easily led.”

[CAREGIVER 8]

Caregivers also revealed potential roadblocks, notably a possible absence of effective communication about the quality of online behaviours between youth and their caregivers. Caregivers can play a significant role in mitigating negative social media influences by regulating their youth’s access to social media, a strategy employed by Caregiver 8:

“This is why we’re trying to get away with not having Facebook for as long as we can… the rule with all my other youth is that if they have got an account I need to be added as a friend… Otherwise, they’re not allowed to use it.”

[CAREGIVER 8]

Research suggests that a measured degree of caregiver control over youth’s social media use can promote healthier interactions [48]. Caregivers’ role in enhancing their youth’s digital connectedness is pivotal, as studies highlight effective caregiver mediation strategies, such as co-using and discussing social media, foster a sense of digital connectedness among youth [49].

Caregivers also recognised their youths’ dependence on them in their social lives. The influence of mental health issues on social dynamics can result in a reliance on caregivers for social experiences, a dynamic that illustrates the complex interplay between the microsystem and mesosystem in the lives of youth [50]).

“He has never had a sleepover at a friend’s house. When he was younger, he always had friends over for sleepovers at our house because he didn’t want to separate from me.”

[CAREGIVER 5]

Lastly, caregivers demonstrated an understanding of their youths’ selective trust dynamics.

“He feels like he connects more to adults than his peer group… I think it’s definitely a trust thing. Like he only talks to one of his friends about what’s really going on.”

[CAREGIVER 4]

Understanding the integral role of social interactions in youth development requires a comprehensive perspective encompassing adult and age-similar relationships. Young individuals often gravitate towards adults, seeking guidance and support, which can be attributed to an inherent desire for acceptance and inclusion [51]. However, for those who have experienced breaches of trust, the pathway to secure attachments can be fraught with obstacles, including social skills deficits and fears of rejection [52].

### 3.2. Theme 2: Supporting Readiness to Engage in Social Relationships

This theme revealed interconnected strategies synergistically promoting youth’s readiness for social engagement. This includes support during social interaction distress, encouragement of social relationships, navigation of mental health symptoms, social skills development, and social problem-solving coaching. In addressing mental health issues among youth, caregiver supporting their youth’s readiness can be a pivotal factor in the recovery process. This assistance aids in social inclusion, thereby enhancing their connectedness [51], and assists in cultivating social skills, particularly beneficial for those who may struggle socially due to their mental health conditions [53]. Caregiver 8 noted her son’s refusal to engage:

“So the biggest problem with [young person] is he will not engage… and I know a child that doesn’t engage is a child that’s crying out for help.”

[CAREGIVER 8]

Caregivers often initiate social contact for their youth:

“I have to pretty much instigate any catch-ups with his peers. He never instigates anything… I think sometimes he wants to connect but doesn’t know how to.”

[CAREGIVER 8]

Research indicates a positive correlation between a caregiver’s ability to understand and respond to their youth’s distress and the youth’s social competence [54]. Caregiver 4 provided additional insights into the challenges of social interaction distress for youth, revealing their struggles with trust, social media use, and peer pressure:

“Well, he has a social media account like he’s got an Instagram account and Facebook, but he doesn’t really talk to people on it… I think it’s definitely a trust thing… there’s been a lot of issues with him developing trust with people.”

[CAREGIVER 4]

Furthermore, Caregiver 4 provided additional insights into the challenges their youth faces due to bullying at school:

“I think he has been teased at school. He’s sort of played it down, but he was on medication that made him gain weight, and he got teased a lot for being fat.”

[CAREGIVER 4]

Research demonstrates caregivers’ that provide supportive and trustful environments can counter the impacts of peer pressure and bullying [55]. Caregivers’ role in preparing their youth for social life, particularly providing them tools to cope with bullying, is crucial. Caregiver 8 describes their youth’s disruptive behaviour and self-harm at school:

“Just losing his [temper] and walking out or stabbing other kids with pencils. But the teacher and the staff there will drop him home because they know when he’s about to have an outburst.”

[CAREGIVER 8]

Supporting youth to reflect on and resolve conflicts at school is a central part of this process. Opportunities for restorative experiences, such as making amends or expressing views following significant incidents, are essential [56,57]. Caregivers play significant roles in managing their youth’s mental health, especially in addressing academic and social difficulties at school, and supporting education staff to understand the influence of mental health on behaviour [58,59]. Caregivers discussed peer support groups and social gatherings as strategies to support and prepare youth’s social engagement:

“Community events… would be really cool to, you know, to meet other people in the community.”

[CAREGIVER 5]

“It did help for her to know that there are other young people who were having similar experiences, but she didn’t want to be in touch with them.”

[CAREGIVER 3]

Peer support groups have been shown to manage youth mental health difficulties effectively. Peer support workers are primarily engaged through mental health services in Australia [60] though can operate through educations systems and other channels also [61]. Peer support workers are generally young adults with lived experience of childhood mental who offer with social-emotional support to youth receiving treatment in mental health services [62]. The emphasis on community engagement and advocating for active youth involvement in their communities fosters positive relationships and psychosocial development [63]. Caregiver 3 speaks to the complex role of peer support groups that provide a shared experience, reducing feelings of isolation, and fostering a sense of belonging [61].

Caregivers must advocate for a consistent and supportive school environment, especially in recovery, to foster readiness to engage in social relationships [64]. Caregivers noted the significance of stable, supportive relationships in the school setting for the wellbeing of youths:

“He couldn’t keep up with the changing of staff every day. Nobody could connect with him. He would come home saying to me that all the teachers did was yell at him all day because, in his head, that’s what he heard.”

[CAREGIVER 8]

Caregivers also talked about the benefits of employment to their youth’s development. Caregiver insights underscore the importance of promoting independence in youth to enhance their participation in social settings. Caregiver 5 shared her concern about her daughter’s entry into the workforce based on their own previous experience with how mental health issues were handled in workplace:

“Well, I think something that I’ve become quite passionate about is [young person] would love to get a job at the moment…and so I have a lot of fear myself about her getting a job and committing to it. You know, I don’t want her first experience to be a bad one… I guess, the intimidation of being in a crowded place, or with people that she doesn’t know.”

[CAREGIVER 5]

The ‘scaffolding’ approach literature reinforces the need for caregivers to find a balance between offering support and fostering independence [65]. Caregiver 5 recognises the need to scaffold her daughter’s transition to employment, being careful not to overprotect.

### 3.3. Theme 3: Promoting Social Problem Solving and Self-Efficacy

Research emphasises the vital role of social problem-solving skills in youth for improving mental health, promoting emotional regulation, reducing negative behaviours, and bolstering mental wellness [66,67,68]. Caregiver 4 shared how the use of music and meditation helps their youth to cope with the noise from other students, indirectly bolstering their social problem-solving competencies:

“He uses music all the time, like when he was going to school, he had, he would use it in the classroom because the noise of other students overwhelms him, like just that background noise…He likes the apps that have like little milestones. Like it’ll tell you on [app], it will tell you when you get to your 10th meditation or whatever.”

[CAREGIVER 4]

Those with a strong sense of self-efficacy tend to engage in positive social behaviours, establish fulfilling relationships, and maintain a strong sense of community belonging [69,70]. This is especially significant given the crucial role that social connectedness plays in fostering mental health resilience [8]. Caregivers noted the importance of enjoyment, autonomy, and personal goals for their youth:

“What’s most important to [young person] is having fun…doing what she wants when she wants.”

[CAREGIVER 1]

“So, it’s really good if she’s got an interest in it, things work better.”

[CAREGIVER 6]

“…and it has to be about her goals as well.”

[CAREGIVER 9]

Goal-directed behaviour can equip individuals to cope with adversities while maintaining self-efficacy. When young individuals experience a sense of control and freedom in their actions, they are more likely to feel competent and connected, boosting their self-efficacy [71]. Caregivers shared their youth’s experience with mental health services, and the critical impacts to their sense of self-efficacy:

“…and then he met [a worker] at [mental health service] last year, and he really connected with him. He seems to be connected really quickly here as well… and …he describes it here as, “I really love it, Mum, that the staff are just interested in us as people like they sit, and they have lunch with us.”

[CAREGIVER 4]

“Teaching her that her psychologist is her safe space. So [young person] goes into all of those sessions by herself. That’s her space. That’s her space to share and have really good discussions.”

[CAREGIVER 9]

These accounts highlight the importance of positive relationships and autonomy in fostering self-efficacy in recovery. Caregiver 9’s emphasis on creating a safe and autonomous space for youth, aligns with the principles of patient autonomy and self-determination in healthcare, which are known to impact self-efficacy positively [72]. Caregivers also help develop youth’s self-efficacy by prompting them to engage socially:

“…and I say to him sometimes, have you messaged any of your friends?…and I’m like, “Do you think you should?”

[CAREGIVER 4]

Caregiver beliefs about their youth’s abilities directly impact the young person’s self-efficacy, influencing their resilience and problem-solving skills.

Caregiver 9 also shared the significance of teaching youth self-regulation strategies:

“But we use zones of regulations particularly for morning routine when we have to get ready for school…So, we encourage as well as possible while she’s feeling some strong emotions or whatnot to look at her toolbox first.”

[CAREGIVER 9]

Caregivers can significantly enhance their youth’s social self-efficacy by creating a supportive environment, embodying effective social behaviour, and promoting social participation.

## 4. Discussion

The escalating prevalence of mental health issues among youth has necessitated the development of innovative, recovery-oriented, and family-centred approaches. Youth occupy a developmental stage that makes them highly vulnerable to social isolation’s adverse effects and associated with mental health issues. The findings of this study revealed the multifaceted roles that caregivers play in supporting the social connectedness and recovery process of youth with mental health concerns. The results of the study, as summarised in Table 3, highlighted three primary caregiver roles: (a) understanding the nature and quality of social networks, (b) supporting readiness to engage in social relationships, and (c) promoting social problem-solving and self-efficacy.

The first theme, understanding the nature and quality of social networks, emphasised the caregiver’s role in comprehending and navigating the youth’s social world. Caregivers assess the youth’s social networks, recognise connection resistance, monitor digital social life, identify and manage dependence on caregivers, and understand trust dynamics. These tasks require caregivers to actively observe and interact with the youth’s social life, understand their emotional needs, and support them in building and maintaining social connections [32,38]. Caregivers as also tasked with supporting youth to prepare for and navigate social interactions as evidenced by the second theme: supporting readiness to engage in social relationships. This includes support during social distress, encouragement of social relationships, navigation of mental health symptoms, development of social skills, and coaching in social problem-solving. Through these tasks, caregivers create a supportive environment that fosters youth’s readiness to engage with others, enhancing their social connectedness [28,32]. The third theme, promoting social problem-solving and self-efficacy, depicts caregivers’ role in building self-confidence and empowering youth to solve social problems independently. Caregivers foster personal goals and interests, encourage autonomy, provide self-regulation strategies, and encourage social participation. These tasks help youth build confidence in their abilities, enhancing their social problem-solving skills and increasing their self-efficacy [20,21].

Existing frameworks recognise caregivers’ influence on youth development but do not specifically address their role in promoting social connectedness among youth with mental health issues [25,32,34,35,73]. Our findings are consistent with the construct of ‘connectedness’ in the CHIME model [25]; however, our research provides a more nuanced understanding of how caregivers promote connectedness, emphasising strategies such as understanding the quality of their youth’s social networks and supporting readiness to engage in social relationships. Moreover, our findings align with the restoration and resilience processes that Dallinger et al. [32] found to be fundamental aspects of youth recovery. Caregivers support restorative processes, such as dealing with adversity and reconnecting with support systems. Caregivers also support resilience processes, such as developing new support networks and teaching self-regulation skills. However, our study goes beyond this by highlighting specific strategies caregivers use to bolster these processes, such as promoting self-efficacy and managing mental health symptoms to enhance social engagement.

These findings may inform the development of a conceptual framework on the roles of caregivers in youth recovery and promoting connectedness by outlining key dimensions of caregiver involvement. The framework could propose that caregiver involvement is a multi-dimensional construct encompassing three primary roles: understanding social networks, supporting readiness for social involvement, and promoting social problem-solving and self-efficacy. Each role is associated with tasks or actions that caregivers undertake to support the youth’s social connectedness and mental health recovery. The framework could also propose that these roles are interrelated and mutually reinforcing, suggesting that effectiveness in one area may enhance effectiveness in others. For example, understanding a youth’s social networks could provide valuable insights that support the readiness to engage in social interactions, which can foster the development of social problem-solving skills and self-efficacy. Moreover, it denotes the challenges caregivers face in fulfilling these roles and the strategies they use to overcome them. These elements could guide the development of interventions and resources aimed at supporting caregivers in their roles and improving recovery outcomes for youth with mental health concerns.

### 4.1. Study Limitations

This study acknowledges several limitations. The sample inadvertently consisted solely of female caregivers from Queensland, Australia, which could limit the generalisability of the findings to male caregivers or caregivers in other geographical regions, ethnicities, and cultures. The extensive experience of these caregivers with mental health services may bias the results, as their more nuanced understanding and developed strategies might not reflect the experiences of those newer to the system. The study’s cross-sectional design offers only a snapshot of a single point in time, failing to capture changes over time in caregivers’ strategies or the youth’s mental health status, an issue a longitudinal study could address. The caregiver–young person relationship is also potentially a key point of connection for the young person. This connection was not explored here as we considered it outside the bounds of this study. Future research should focus upon issues involved promoting and strengthening the caregiver relationship for young people. Lastly, the study’s focus on youth with severe mental health issues may not apply to caregivers of youth with milder mental health issues who face different challenges and employ distinct strategies. These limitations underscore the necessity for further research.

### 4.2. Implications for Policy, Practice, and Future Research

Caregivers are vital in promoting social connectedness among youth with mental health issues [28]. Their roles encompass understanding social networks, advocating social engagement, symptom management, social skill enhancement, and social problem-solving coaching [10,22]. Caregivers need to be empowered with relevant knowledge and tools, recognising their crucial role in supporting young people [32]. Caregivers’ encouragement and introduction of strategies like utilising music and meditation apps, promoting community group involvement, and encouraging daily kindness acts are supportive of the development of self-efficacy and social problem-solving skills [11,74]. These techniques could be incorporated into youth recovery interventions. Caregivers encounter challenges like managing digital social interactions, the effects of mental health symptoms on social interplay, and teaching social problem-solving complexities [32,74]. These insights could enable mental health practitioners to customise their aid to caregivers, mitigating these obstacles and enhancing their capacity to foster social connectedness in youth during recovery.

The study highlights the critical need for policies that strengthen the caregiver’s role in youth mental health recovery including the provision of necessary resources, fostering inclusiveness, and the development of socially connected environments in schools and communities. These policies must also enhance the safe usage of digital platforms for social connection by reducing cyberbullying and promoting digital literacy among caregivers. Caregivers described various challenges they face in promoting connectedness but did not provide explicit strategies for overcoming them. The lack of strategies for some challenges highlights an area requiring further investigation. It indicates a need for more research focused explicitly on equipping caregivers with evidence-based methods to tackle hurdles in promoting connectedness. This knowledge gap allows future studies to build on these initial findings and delve deeper into developing practical solutions that caregivers can employ.

Overall, the findings reveal that while caregivers already utilise various skills in supporting connectedness, they require additional evidence-based tools to address some persistent challenges. Systemic policies and mental health treatment centres should encompass caregiver education and ongoing support, while also promoting inclusivity and targets for the reduction of mental health stigma. Directed research focused on filling these gaps through co-design and testing of practical solutions could significantly empower caregivers and improve outcomes for youth mental health recovery.

## Figures and Tables

**Table 1:** Demographic information of youth in care of participants.

Reported Gender	Age	Residential Location
Female	14	Metropolitan
Male	15	Metropolitan
Female	16	Regional
Nonbinary	16	Metropolitan
Female	17	Metropolitan
Female	15	Metropolitan
Trans female	15	Rural or remote
Nonbinary	15	Metropolitan
Female	15	Metropolitan

**Table 2:** Participant Demographic Information.

Caregiver	Age	Location	Education	Time Supporting Youth in MHS
1	46	Metro	Post-Graduate	9 years
2	49	Rural	Year 12	2 years
3	40	Regional	Diploma	4 years
4	53	Metro	Bachelor’s Degree	4 years
5	44	Metro	Bachelor’s Degree	4 years
6	45	Metro	Certificate	2.5 years
7	50	Metro	Year 11	7.5 years
8	44	Rural	Associate Diploma	5 years
9	42	Regional	Bachelor’s Degree	2 years

Note. Time Supporting Youth in MHS = Number of years caregiver has been supporting youth while they are engaged with mental health services; Metro = Metropolitan; Education = Highest level of education undertaken.

**Table 3:** Summary of Findings from Thematic Analysis.

Theme (Caregiver Role)	Task of Caregiver	Purpose	Challenges Faced	Strategies to Overcome Challenges
Understanding the Nature and Quality of Social Networks	Assess youth’s social networks	Comprehend social experiences and needs	Reluctance to share, understanding digital impacts	-
	Recognise connection resistance	Identify and overcome social barriers	Youth resistance, mental health issues	Use of mental health services, peer support
	Monitor digital social life	Ensure healthy online interactions	Resistance, understanding digital nuances	Rules for social media use
	Identify and manage dependence on caregivers	Foster independence with support	Balancing support and independence	-
	Understand trust dynamics	Facilitate secure attachment	Trust breaches, fostering trust	-
Supporting Readiness to Engage in Social Relationships	Support during social distress	Help navigate social discomfort	Reluctance to engage, interpreting distress	Open communication
	Encourage social relationships	Facilitate social interactions	Distrust, difficulty initiating catchups	Initiate interactions on behalf of youth
	Navigate mental health symptoms	Equip with coping strategies	Misunderstandings, stigma, lack of support	Advocacy, coordinate with school staff
	Develop social skills	Enhance interaction abilities	Potential skills deficits due to mental health issues	Encourage acts of kindness
	Coach social problem-solving	Build resilience	Difficulty handling conflicts, stressors	Teach conflict resolution
	Support employment engagement	Foster independence, competence	Fear of negative experiences, anxiety	-
Promoting Social Problem Solving and Self-Efficacy	Foster personal goals and interests	Enhance self-efficacy, motivation	Struggle to identify or understand goals	-
	Encourage autonomy	Enhance sense of control	Balancing support and autonomy	-
	Provide self-regulation strategies	Aid in managing emotions and behaviours	Resistance to learning strategies	Teach self-regulation strategies
	Encourage social participation	Enhance social self-efficacy	Resistance due to anxiety or other issues	Prompt social interactions
	Use tools (e.g., music) to manage stress	Enhance focus, self-efficacy	Identifying suitable tools	Use of music as a tool

Note. Empty entries for ‘Strategies to Overcome Challenges’ indicate caregivers provided no strategies.

## Data Availability

The data analysed during the current study are not publicly available due to client confidentiality but are available from the corresponding author on reasonable request although restrictions apply to the availability of these data.

## Supplementary Materials

The following supporting information can be downloaded at: https://www.mdpi.com/article/10.3390/children11121469/s1, File S1: The Role of Caregivers in Promoting Connectedness in Youth with Mental Health Concerns.
